# Computational classifiers for predicting the short-term course of Multiple sclerosis

**DOI:** 10.1186/1471-2377-11-67

**Published:** 2011-06-07

**Authors:** Bartolome Bejarano, Mariangela Bianco, Dolores Gonzalez-Moron, Jorge Sepulcre, Joaquin Goñi, Juan Arcocha, Oscar Soto, Ubaldo Del Carro, Giancarlo Comi, Letizia Leocani, Pablo Villoslada

**Affiliations:** 1 Department of Neuroscience, CIMA-University of Navarra, Pamplona, Spain; 2 Department of Neurology, Clinical Neurophysiology and Neurorehabilitation, University Vita-Salute, Scientific Institute Hospital San Raffaele, Milan, Italy; 3 Center for Neuroimmunology, IDIBAPS-Hospital Clinic, Barcelona, Spain

## Abstract

**Background:**

The aim of this study was to assess the diagnostic accuracy (sensitivity and specificity) of clinical, imaging and motor evoked potentials (MEP) for predicting the short-term prognosis of multiple sclerosis (MS).

**Methods:**

We obtained clinical data, MRI and MEP from a prospective cohort of 51 patients and 20 matched controls followed for two years. Clinical end-points recorded were: 1) expanded disability status scale (EDSS), 2) disability progression, and 3) new relapses. We constructed computational classifiers (Bayesian, random decision-trees, simple logistic-linear regression-and neural networks) and calculated their accuracy by means of a 10-fold cross-validation method. We also validated our findings with a second cohort of 96 MS patients from a second center.

**Results:**

We found that disability at baseline, grey matter volume and MEP were the variables that better correlated with clinical end-points, although their diagnostic accuracy was low. However, classifiers combining the most informative variables, namely baseline disability (EDSS), MRI lesion load and central motor conduction time (CMCT), were much more accurate in predicting future disability. Using the most informative variables (especially EDSS and CMCT) we developed a neural network (NNet) that attained a good performance for predicting the EDSS change. The predictive ability of the neural network was validated in an independent cohort obtaining similar accuracy (80%) for predicting the change in the EDSS two years later.

**Conclusions:**

The usefulness of clinical variables for predicting the course of MS on an individual basis is limited, despite being associated with the disease course. By training a NNet with the most informative variables we achieved a good accuracy for predicting short-term disability.

## Background

Multiple sclerosis (MS) is a clinically heterogeneous disease and its course in an individual patient is largely unpredictable. The failure to reach an accurate prognosis makes clinical management difficult; this represents one of the most disturbing aspects of the disease perceived by the patients [1]. In order to provide an accurate prognosis during the early or mid-phase of the disease, as well as to monitor both disease course and response to therapy, there is a need to define adequate clinical or biological markers that may serve as surrogate end-points [2]. To date, several clinical variables have been associated with differences in disease outcome [3-5]. In addition, neuroimaging studies [6-8], quantification of axonal loss in the retinal nerve fiber layer [9] or serum and cerebrospinal fluid markers [10] seem to be associated with disease prognosis. However, these clinical and biological markers, even though they show statistical correlation with clinical end-points, are limited in predicting the disease course on an individual patient basis due to their low *diagnostic accuracy *(sensitivity, specificity, positive predictive value, negative predictive value, area under the ROC curve-AUC-and accuracy) or to the lack of information about their performance and robustness in multicenter studies. From a clinical perspective, markers of disease activity (relapses or disability) should be straightforward, cost-effective and capable of being standardized in clinical settings.

Computational classifiers, such as neural networks (NNets), Bayesian networks, linear regression models or decision-trees, are mathematical algorithms that maximize the matching between the input data and the output (prediction), becoming very useful in the field of Biomedical Informatics. They can extract more information from complex dataset without the need to adjust to a linear model (except for the regression model). Also, they can accommodate prior information and achieve higher accuracy for predicting outcomes. Therefore, these classifiers are promising tools for providing valuable insights about complex diseases, such as disease prognosis or response to therapy. Here, we took advantage of several computer-assisted support systems that can filter and integrate complex medical data as well as provide helpful clinical answers at the level of the individual patient [11].

Validation of clinical biomarkers requires conducting prospective studies, adhering to the STARD criteria, and evaluating their diagnostic accuracy for predicting the end-points [2]. In order to become clinically useful in the decision-making process at the individual patient level for both the patient and the physician, a biomarker should have a high accuracy, above 90% at least. Due to the heterogeneity and dynamic nature of complex diseases, achieving a high accuracy for a given biomarker is challenging. However, promising results are currently being obtained in cancer and other multifactorial disorders through the use of combined biomarkers and clinical information in a computational classifier such as a NNet [11].

**Figure 1:**
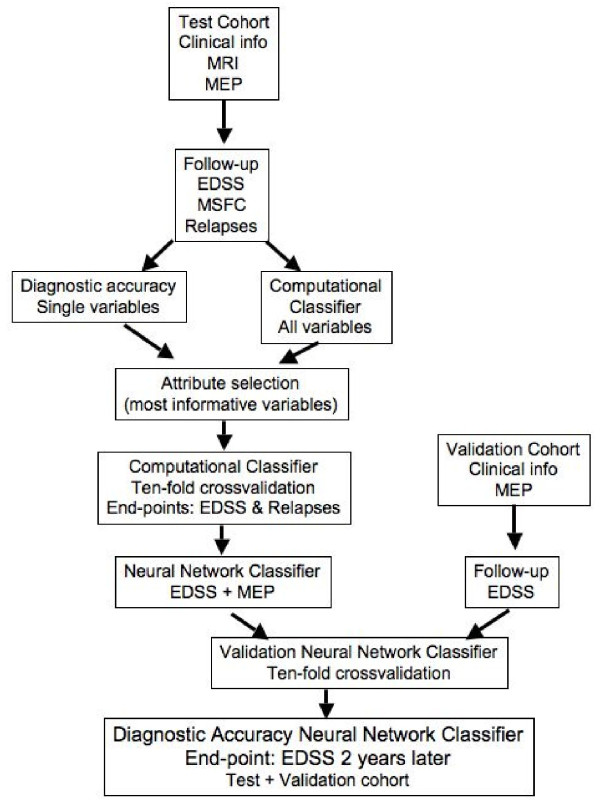
**Flow-chart of the study**. We obtained clinical data, MRI and MEP metrics from the test cohort. The test cohort was followed for two years, collecting clinical information (disability and relapses). Single variables were tested for predicting disease activity (new relapses or increase in the disability scales EDSS or MSFC) outcomes and predictive models were developed using computational classifiers, after performing an attribute selection of the most informative variables. The different classifiers were tested in the test cohort using a 10-fold cross-validation. From the different variables and classifiers, the NNet using EDSS at baseline and CMCT for predicting the EDSS range two years later was selected for further development because of its high performance. Validation was carried out in a second prospective cohort for whom EDSS at baseline and two years later and CMCT were available. Finally, we calculated the diagnostic accuracy of the NNet using the 10-fold cross-validation method in the overall population (test and validation cohorts).

The aim of this work was to evaluate the usefulness of clinical, imaging and neurophysiological variables for predicting short-term disease outcomes in MS patients. Based on published studies, we expected that single biomarkers would not achieve enough accuracy (AUC ≥ 90%) for predicting clinical end-points, therefore we aimed to develop predictive models by incorporating combined clinical information in different computational classifiers that could offer a high AUC for predicting future disability of individual MS patients in multicenter settings (Figure 1).

## Methods

### Test cohort

We prospectively recruited 51 consecutive patients with MS [12] at the University of Navarra, Spain. Patients with clinically isolated syndrome (CIS) fulfilled the criteria of spatio-temporal dissemination [12]. In order to select a cohort of patients in the early-mid phase of the disease for whom predicting disease activity would be more valuable, the proposed inclusion criteria were as follows: short-medium disease duration (< 10 years), any disease subtype and no relapses in the month prior to inclusion. The use of immunomodulatory therapy was permitted. The exclusion criteria were those conditions that prevent patients from undergoing motor evoked potentials (MEP) or MRI studies and subjects with EDSS > 7.0. Once study approval from the Institutional Review Board (IRB) was obtained, patients were included after giving their informed consent.

Neurological examination and disability assessment were performed quarterly over a 2-year period. Trained personnel scored physical disability using the expanded disability status scale (EDSS) [13], the MS severity scale (MSSS) [14] and the MS functional composite (MSFC) [15]. The EDSS by the end of the study was confirmed in a second visit 6 months later and was categorized in three intervals: mild (0-2.0), moderate (2.5-4.5), severe (≥ 5.0). The MSFC included the timed walked test (TWT), the nine-hole peg test (NHPT) and the paced auditory serial addition test (PASAT). We adopted the definition of disability progression as a change of ≥ 1 point in the EDSS (≥ 0.5 for those with baseline EDSS of 6.0 or 6.5), being confirmed in a second visit 6 months later [16]. None of the patients refused to undergo neurological examination, MEP or MRI studies at the time of entry into the study. Data collection was planned prior to performing the tests. The baseline characteristics of the patients are shown in Table 1.

**Table 1:** Demographics at baseline and clinical variables at baseline and follow-up of the MS patients and the control group of the test cohort

	MS	HC	p value
N	51	20	--
Age	35.1 (8.9)	33.2 (9.1)	n.s.
Sex (male/female)	18/33	5/15	n.s.
Duration (years)	5.9 (7.4)	--	--
Disease subtype	CIS (16), RRMS (26), SPMS (3), PPMS (4), PRMS (2)	--	--
Number of relapses in the previous 2 years	1.29 (1.51)	--	--
EDSS at baseline	2.0 (0-6)	--	--
MSSS at baseline	4.6	--	--
MSFC at baseline	0.08 (0.70)	--	--
EDSS at follow-up	2.0 (0-7)	--	--
Change EDSS at follow-up	0.35 (0.37)	--	--
Disability progression at follow-up (yes/no)	15/36	--	--
MSFC at follow-up	0.19 (0.67)	--	--
Relapse rate follow-up	0.57 (0.50)	--	--
Relapse-free patients follow-up	21	--	--
DMD (yes/no)	28/23	--	--
DMD type (Avonex/Betaferon/Rebif/Copaxone/Azatioprine/Mitoxantrone)	3/8/9/0/1/1	--	--
Right ADM CMCT (ms)	10.63 (3.88)	8.09 (1.22)	0.009
Left ADM CMCT (ms)	10.71 (3.73)	7.91 (1.25)	< 0.0001
Right FHB CMCT (ms)	22.64 (10.57)	14.74 (2.36)	< 0.0001
Left FHB CMCT (ms)	20.52 (6.82)	15.81 (3.14)	< 0.0001
T1 lesion volume (cm^3^)	16.72 (20.23)	--	--
T2 lesion volume (cm^3^)	47.56 (42.77)	--	--
Gad+ volume (cm^3^)	0.29 (0.94)	--	--
GM volume (cm^3^)	522.50 (52.29)	--	--
WM volume (cm^3^)*	506.38 (59.40)	--	--

The results are described as the mean (SD), except for the EDSS, which is expressed as the median (range). Disability progression: increase of 1 point in the EDSS confirmed at 6 months (≥ 0.5 for those with baseline EDSS of 6.0 or 6.5). MEP and MRI variables were obtained at the time of study inclusion (baseline assessment).

HC: Healthy control; MS: Multiple sclerosis; CIS: Clinically isolated syndrome; RRMS: Relapsing-remitting MS; SPMS: Secondary-progressive MS; PPMS: Primary-progressive MS; PRMS: Progressive-relapsing MS; EDSS: Expanded disability status scale; MSSS: MS severity score; MSFC: MS functional composite; DMD: Disease modifying drugs; CMCT: Central motor conduction time; ADM: Adductor digiti minimi; FHB: Flexor hallucis brevis; ms: Milliseconds; Gad+: Gadolinium-enhancing lesions; GM: Grey matter; WM: White matter (* excluding lesions).

### Second cohort

A second independent cohort was recruited for validation purposes at the Hospital San Raffaele (Milan, Italy). This group included 96 MS patients (age: 37 ± 10 years; sex: 32 male and 64 female; disease duration: 9 ± 6 years; median EDSS at baseline: 1.5, range: 0-6.5). In this cohort, 85 patients had RRMS and 11 SPMS. The patients were followed prospectively for two years by trained neurologists who obtained the EDSS at the end of the second year of follow-up. After 2-year follow-up, the median EDSS score and the mean EDSS change were 2.0 (range: 1.0-6.5) and 0.3 ± 0.8, respectively. Additionally, a MEP study was performed at the baseline (Table S4) [17]. The local IRB approved this study and the patients were recruited by their neurologists after obtaining written informed consent.

### Motor Evoked Potentials

We measured the following MEP parameters: motor threshold, area, amplitude, latency, central motor conduction time (CMCT), and silent period (SP) with and without facilitation. MEP amplitudes and latencies were considered abnormal if they differed in ≥ 2.5 SD from the normative database of our center; amplitude was also regarded as abnormal if there was a side-to-side difference of ≥ 50%. CMCT was calculated by subtracting the peripheral conduction time, measured using the F-wave method, from the central latency [18]. Evoked potential abnormalities were quantified for each limb according to a scale modified from Leocani et al. [17] (0 = normal, 1 = increased latency, 2 = increased latency plus decreased amplitude, 3 = absence of MEP response); a MEP score involving the 4 limbs was established ranging from 0 to 12. To analyze the effect of asymmetric disability, a Z score was created for each limb using the CMCT (Z = (CMCT-mean)/SD); the worst Z score of the 4 limbs was selected and compared with disability and disease subtype. MEP studies were performed by a trained neurologist (OS, JA, LL, MB, UDC), blinded to clinical and MRI data.

### MRI studies

At the time of the first visit, MRI studies were performed on a 1.5 T Magnetom Symphony Maestro Class (Siemens, Erlangen, Germany) as described elsewhere [9]. We used MRIcro software (http://www.cabiatl.com/mricro/mricro/mricro.html) to manually delimit the lesions in the T1-and T2-weighted scans of all patients (intraclass correlation coefficient, rI = 0.892; p < 0.001) [19]. In order to quantify the grey (GM) or white matter (WM) volume, a voxel-based morphometry (VBM) analysis of the 3D T1-weighted studies was performed using the SPM2 software. To obtain the normalized and segmented images of each subject but avoiding the bias introduced by WM lesions, we employed a modified protocol of the improved VBM method optimized for MS as described elsewhere [20]. A trained neurologist (JS), blinded to the MEP results, carried out the MRI analysis.

### Statistical analysis

The statistical package for the social sciences (SPSS Inc., Chicago, IL, USA) version 13.0 was used for the analysis, setting the significance level at 0.05. From among the four MEP measurements of every subject we only considered the most pathological result for each patient to calculate the predictive value of the MEP test. The diagnostic accuracy of the different tests was assessed by using sensitivity, specificity, positive and negative predictive values, AUC, as well as the 95% confidence intervals (CI). According to some web-based interactive statistics software (http://statpages.org/), the accuracy (A) is calculated by dividing the number of true positives (TP) and true negatives (TN) by the number of positives (P) and negatives (N), that is, A = (TP + TN)/(P + N). To determine the strength of agreement, we computed Cohen's kappa statistic as a chance-adjusted measure of agreement between observers: poor (< 0.00), slight (0.00-0.20), fair (0.21-0.40), moderate (0.41-0.60), substantial (0.61-0.80), and almost perfect (0.81-1.00) [21,22].

### Computational classifiers

We made use of several deterministic classifiers: 1) Naïve Bayes; 2) Simple logistic (an up-to-date representative of logistic regression models that uses boosting to calculate the regressions); 3) Ramdom decision-tree meta-classifier. We also investigated non-deterministic classifiers, such as the multilayer perceptron (MLP), which is one of the most widely used NNets. All these algorithms were implemented in WEKA (Waikato Environment of Knowledge Analysis) v 3.5.8, (http://www.cs.waikato.ac.nz/ml/weka). Classifier performance was quantified according to the AUC as follows: excellent (90-100%), good (80-90%), intermediate (70-80%), and fair (< 70%) [21,22].

Because of the negative effect of unhelpful attributes on most machine learning schemes, we followed a learning procedure that incorporated an initial feature selection stage (data mining), which strives to eliminate all but the most relevant attributes [23]. For attribute selection, we chose the Wrapper approach, which evaluates the suitability of each attribute subset by estimating the accuracy of the specific classifier used, since it is more consistent and shows better predictive accuracy than filter approach, in which the features are filtered independently of the induction algorithm or classifier [24,25].

The different classifiers were tested using a 10-fold cross-validation which partitions the original sample into 10 sub-samples. Of these 10 subsamples, a single sub-sample was retained as the validation data to test the model while the remaining 9 sub-samples were used as training data [26]. The cross-validation process was then repeated 10 times with each of the 10 sub-samples being used once as the validation data, and the 10 results obtained could then be averaged to produce a single estimate. To seek an accurate error estimate, the above 10-fold cross-validation process was repeated 10 times (the learning algorithm is invoked 100 times on datasets that are all nine-tenths the size of the original) and the results were averaged obtaining a mean square error (MSE). During the training process of the neural network cross-validation was used for detecting when overfitting starts; then training was stopped before convergence (automatic early stopping) to avoid overfitting. Though there are a number of plausible stopping criteria, we stopped the training when the validation set reached the minimum MSE [27].

### Attribute selection and validation procedure

As inputs, we considered the following attributes: a) clinical variables: disease subtype, sex, age, EDSS at study entry, motor function score of EDSS (MF), MSFC, motor scores of MSFC (TWT and NHPT), use of disease modifying therapies; b) MRI variables: total lesion volume on T1, T2 or gadolinium-enhancing T1, GM and WM volumes; c) neurophysiological variables: CMCT, MEP score, aggregated MEP score, worst Z score from the 4 limbs, abnormal MEP. First the selection process for each classifier ranked the attributes using the Wrapper algorithm, and then we chose the ones with the highest ranks as selected inputs to the corresponding classifier. As the primary end-point (output or dependent attribute) for each classifier, we considered the EDSS change at the end of the study (confirmed after six months), since this is the most common end-point in MS clinical trials assessing the efficacy of disease modifying drugs. Secondary outcomes were disability progression (yes or no), and the occurrence of relapses by the end of the study (relapse-free status).

The different classifiers were tested in the test cohort using a 10-fold cross-validation, because this provides the best method for testing classifiers [26]. Subsequently, we also validated our findings applying the same classifiers to the second cohort from Italy for whom EDSS at baseline and two years later and CMCT were available. Finally, we calculated the diagnostic accuracy of the NNet (MLP) using the 10-fold cross-validation method in the overall population (test and validation cohorts).

*See *additional file 1* for more detailed information of the procedures*.

## Results

### Diagnostic accuracy of clinical, neuroimaging and neurophysiological variables in predicting short-term disease activity

We found that disease subtype (progressive forms), sex (male) and EDSS at baseline had fair-to-intermediate diagnostic accuracy to predict EDSS change or disability progression two years later (Table 2). No clinical variable was considered as very accurate to predict the other disease outcomes during the follow-up period.

**Table 2:** Diagnostic accuracy of single clinical, imaging and MEP variables for predicting short-term disease activity in the test cohort

Clinical end-points	Change EDSS	Disability Progression	Relapse free
MS subtype	64 (8) < 0.01	70 (9) < 0.01	54 (17) 0.03

Sex	62 (11) -0.02	70 (9) < 0.01	67 (21) 0.30

Age	68 (15) 0.22	69 (10) -0.01	57 (16) 0.03

Baseline EDSS	65 (16) 0.18	72 (13) 0.16	59 (11) 0.07
Baseline MF	70 (17) 0.28	71 (14) 0.16	59 (15) 0.09
Baseline MSFC	63 (10) < 0.01	67 (12) -0.02	58 (6) -0.02
Baseline TWT	63 (12) 0.03	71 (11) 0.05	57 (5) -0.03
Baseline NHPT	66 (14) 0.16	64 (13) -0.06	58 (10) 0.03

Treatment (DMD)	63 (11) -0.02	70 (10) 0.00	57 (8) -0.02

CMCT ADM	64 (8) < 0.01	70 (10) < 0.01	55 (14) -0.02

CMCT FHB	69 (15) 0.21	75 (15) 0.28	58 (4) -0.01

MEP score	59 (10) -0.05	67 (13) -0.01	56 (8.07) -0.04

Grouped MEP score	64 (15) 0.10	71 (12) 0.10	55 (9) -0.05

CMCT Z score	65 (14) 0.15	70 (15) 0.17	58 (5) -0.01

Abn TMS	59 (12) 0.01	66 (11) < 0.01	57 (7) -0.02

T1 vol	63 (10) -0.03	70 (9) < 0.01	56 (12) 0.01

Gad+ vol	64 (8) < 0.01	70 (9) < 0.01	58 (5) -0.01

GM vol	63 (11) -0.02	69 (10) -0.01	61 (17) 0.14

WM vol*	64 (13) 0.06	69 (11) -0.02	61 (18) 0.16

Results are expressed as the Accuracy (%), standard deviation (SD) and Kappa value (in decimals) of the test cohort using the Simple Logistic algorithm. CMCT ADM and CMCT FHB refer to the average CMCT of left and right arm or leg, respectively.

EDSS: Expanded disability status scale; MF: Motor score of the EDSS; MSFC: MS functional composite; TWT: Fastest time in seconds to walk 25 feet; NHPT: Nine hole peg test; DMD: Disease modifying drugs; CMCT: Central motor conduction time; CMCT ADM: Worst CMCT of both arms (ADM: Adductor digiti minimi) at baseline; CMCT FHB: Worst CMCT of both legs (FHB: Flexor hallucis brevis) at baseline; MEP score: Combined MEP score for the four limbs; Grouped MEP score: MEP score grouped in 3 classes (1 for 1-3, 2 for 4-6, and 3 for 7-9); CMCT Z score: Worst CMCT Z score of the four limbs at baseline; Abn TMS: Abnormal MEP (presence or absence of at least one abnormal MEP in each patient); T1 vol: Lesion volume in T1 at baseline; Gad+ vol: Gadolinium-enhancing lesion volume at baseline; GM vol: Grey matter volume at baseline; WM vol: White matter volume at baseline (* excluding lesions).

Regarding neuroimaging and MEP variables, first we assessed their correlation with EDSS both at baseline and follow-up in order to gain some insight about their association with permanent disability. Then, we tested their diagnostic accuracy for predicting disease outcome. Several MRI abnormalities correlated moderately with EDSS at baseline, such as the lesion volume on T1 and T2 and GM volume (Table 3). However, GM volume was the only MRI measure that correlated significantly with EDSS two years later (r = -0.377, p = 0.007). Additionally, MRI variables were considered to have intermediate diagnostic accuracy to predict disease end-points, being T1 lesion load, and GM or WM atrophy the best neuroimaging predictors of disability progression (Table 2).

**Table 3:** Correlation between baseline MRI variables, CMCT maximum Z score and MEP score and the EDSS both at baseline (month 0) and by the end of the follow-up (month 24) in the test cohort

	Baseline (month 0)		End of follow-up (month 24)	
	**r**	**p**	**r**	**p**

Number of lesions in T2	0.327	0.017	0.128	0.05
Lesion volume in T2	0.357	0.029	0.070	0.05
Number of lesions in T1	0.402	0.003	0.174	0.05
Lesion volume in T1	0.413	0.002	0.169	0.05
Number of Gad+ lesions	0.006	0.05	0.143	0.05
Gad+ lesions volume	-0.007	0.05	0.138	0.05
GM vol	-0.368	0.007	-0.377	0.007
WM vol	-0.120	0.05	-0.014	0.05
Whole WM vol	-0.000	0.05	-0.082	0.05
CMCT max-Zscore	0.497	0.001	0.441	0.001
MEP score	0.515	0.001	0.472	0.001

Gad+: Gadolinium-enhancing lesions; GM vol: Grey matter volume at baseline; WM vol: White matter volume at baseline; Whole WM vol: WM vol + lesion volume; CMCT max-Zscore: Central motor conduction time maximum Z score.

With respect to neurophysiological variables (see additional file 1, Fig. S1), we found that the MEP score was significantly correlated with EDSS by the end of the follow-up (Table 3, Table S1 and Fig. S2), yet it was not correlated with the MSFC. By contrast, the CMCT Z score was closely correlated with both EDDS and MSFC at follow up. Furthermore, when comparing the different disease subtypes, the CMCT Z score in CIS and RRMS was correlated with EDSS (baseline EDSS, r = 0.428, p = 0.004; EDSS at month 24, r = 0.338, p = 0.029). Indeed, abnormal motor evoked potentials (CMCT Z score ≥ 2.5, or MEP score ≥ 1) were associated with disease progression (p < 0.001, and p = 0.009, respectively). Finally, several MEP variables displayed intermediate diagnostic accuracy to predict disability progression (Table 2).

Overall, neither the clinical variables obtained at study entry nor the MRI variables nor the MEP studies could be considered as good or excellent in terms of their diagnostic accuracy to predict disease activity (Table 2). Although some of these variables showed an accuracy higher than 70%, the quality of the predictor as defined by the kappa value was low. For this reason, we decided to combine all variables and to profit from the advantages offered by computational classifiers (naïve Bayes, simple logistics, random decision-tree classifiers, NNets) in order to obtain a tool with good accuracy for predicting disease end-points.

### Building computational classifiers to predict disease outcome and valiation in an independent cohort

The diagnostic accuracy to predict the clinical end-points using different classifiers when all the attributes were used is summarized in Table S2. Although the NNet classifier was relatively accurate for some end-points, the majority fell below 70%, which makes them less useful from a clinical point of view. Hence, in order to increase the accuracy rate to predict the different outcomes we performed an attribute selection of the most informative variables for each classifier (Table S3). After selecting the attributes, most classifiers achieved a higher diagnostic accuracy, obtaining the best results with NNets for predicting EDSS change (accuracy = 80%; Table 4). However, all classifiers, including the NNets, were associated with a more modest accuracy and a lower kappa value when predicting the occurrence of relapses (relapse-free) (Table 4).

**Table 4:** Diagnostic accuracy of NNets for predicting clinical end-points after attribute selection in the test cohort

	End-points	A% (SD)	S %	Sp %	NPV %	AUC% (SD)	PPV %	Kappa
	Change EDSS	80 (14)	92	61	80	76 (25)	80	0.54
NNets	Disability progression	75 (17)	87	52	61	74 (31)	80	0.37
	Relapse-free	67 (21)	53	77	70	65 (22)	61	0.33

The results are expressed as the percentage (SD). The attributes selected for the different classifiers (see Table S4) were: 1) Clinical variables: disease subtype, age, sex, EDSS, motor score of the EDSS, MSFC, NHPT, TWT; 2) MRI variables: T1 lesion volume, Gad+ lesion volume, GM volume, WM volume; 3) MEP variables: presence of pathological MEP, MEP score, CMCT.

SD: Standard Deviation; AUC: Area under ROC curve; A: Accuracy; S: sensitivity; Sp: specificity; PPV: positive predictive value; NPV: negative predictive value.

Our results indicated that the most accurate classifier was the NNet for predicting the EDSS change two years later. Moreover, we found that EDSS and MEP variables were the most informative attributes (Table 2) and were always selected by the attribute selection process for all algorithms (Table S3). Accordingly, we decided to validate the NNet in an independent prospective cohort from a second center. As inputs to the classifier, we included only the most informative ones as explained above: a) EDSS at study entry; b) MEP variables: CMCT and abnormal MEP (yes or no). We set the EDSS change at the end of the study (month 24) as the output (dependent attribute). The performance of the NNet classifier in the second cohort was similar to that of the test cohort (accuracy = 81%).

## Discussion

In this study we set out to assess the diagnostic accuracy of clinical, imaging and MEP variables to predict short-term disease activity (increase in the EDSS or new relapses) in MS. As expected, we found that no individual variable was capable of accurately predicting the development of sustained disability or the onset of a relapse during the 2-year follow-up. Consequently, we developed computational classifiers that were able to capture the most valuable information to predict the required outcome (e.g., EDSS or presence of relapses).

The critical issue in developing a NNet is generalization, that is, ability to make predictions for cases that are not in the training set. A NNet that is too complex or excessively trained may fit the noise leading to overfitting, which can result in predictions that are far beyond the range of the training data (poor predictive performance) [28]. One way to avoid overfitting is to use more training cases than weights in the network. Since our cohort does not have a great number of patients, we needed to minimize the complexity of the network (less number of neurons and weights), which is achieved through a reduction of the dimensionality of the network by considering only the most relevant attributes through a feature subset selection. This process is complex and remains an important issue in statistics and many other domains [29]. As feature selection approach we chose the wrapper method because the learning algorithm is wrapped into the selection procedure. Furthermore, since NNets are non-linear systems, which do not make use of explicit parametric hypothesis, selection methods must remain computationally feasible for being useful; so, we did not consider bootstrap, based on the statistical procedure of sampling with replacement, at each step of the selection because it would be very computer intensive [29].

A well-known strategy to prevent overtraining during cross-validation is automatic early stopping through monitoring the mean square errors (MSE) of both the training and validation set [27]. The MSE of the training set decreases as long as the training proceeds. However, the MSE of validation set decreases during the first phase of training, then reaches a minimum value and subsequently starts to increase. In order to avoid overfitting we stopped the training process when the validation set reached the minimum MSE.

It is striking that the only outcome for which NNets were able to obtain a good accuracy was the change in the EDSS. These results highlight the difficulties in predicting the short-term prognosis in complex diseases such as MS. It is noteworthy that none of the classifiers tested was particularly accurate when clinical variables (mainly the EDSS) were not included. This may be because the primary end-point was to predict the change in the EDSS two years later, and by including the EDSS at baseline we provided the classifiers with an approximation to the requested outcome. The inclusion of the EDSS was not an a priori decision, but a result of the attribute selection process. It could be argued that by including the variable to be predicted (outcome) in the list of predictors we might have introduced a bias. Although this could have some potential impact on the validity of statistical tests, it is not affecting the different classifiers, including the one with the best performance, such as NNets. This is because they are not based on rejecting a null hypothesis but on weighting different levels of evidence (information) for matching input variables and output variables (end-points).

Both cohorts were composed of patients at the early to mid-phase of the disease, at a time when having a prognosis is most valuable in influencing therapeutic decisions. Moreover, they do not have a very active disease, which is in agreement with recent clinical trials and prospective studies, in part because they were treated with disease modifying drugs (DMD). Although this represents a greater challenge for any classifier, this is a common scenario in present clinical practice. Indeed, the majority of our patients had a low EDSS score, which implies little disability and imposes more difficulties to predict future disability, as was the case for the MSSS or the MSFC. Even then, the classifiers worked better after including the EDSS at baseline, suggesting that small differences in the EDSS early in the disease might have important consequences in the long-run. However, incorporating other measurements of disability, such as the MSFC and the MSSS, into the classifiers for predicting the same measurements 2 years later did not provide good classification results; this indicates that the use of the outcome variable as part of the predictor does not guarantee an excellent performance.

The classifiers tested worked better after combining the clinical information with several MRI and MEP variables. The MRI variable that most contributed to predicting future disability was GM atrophy, although its weight in the attribute selection process was lower than that of EDSS and MEP. This is in agreement with recent findings suggesting that GM damage seems to be one of the most critical factors leading to MS disability [30]. It is striking that some other MRI variables that are also considered surrogate end-points of disease activity in clinical trials (T1 and T2 lesion load or WM atrophy) did not provide useful information to forecast future disability, even though they were correlated with disability at baseline. Although in our study the validated classifier did not include MRI variables because of the aforementioned reasons, enhanced classifiers could incorporate GM atrophy or other new MRI metrics.

We paid special attention to the MEP variables because they are highly sensitive to injury of motor pathways, and as such they are closely correlated with the EDSS score [31]. We found that the most informative MEP variable was the CMCT, although the MEP score also provided valuable information. Central conduction latencies calculated with the CMCT are very sensitive to demyelination of motor pathways, as well as to axonal loss. Surprisingly, some other measures like MEP amplitude or silent periods were not as informative, even though they seems to be closely related to axonal loss.

The NNet classifier using baseline EDSS and CMCT was able to predict the change in the EDSS two years later with good accuracy. However, its ability to predict some other outcomes such as disability progression, change in the EDSS or the MSFC was not as good. This could be due to the nature of such scores, such as the qualitative nature of the disability progression end-point or the multidimensional character of the MSFC. In addition, the poor capacity of the classifiers to predict the occurrence of relapses (relapse-free) or the number of relapses during the follow-up represents a major challenge, even when the classifiers incorporate several variables associated with relapses, such as the number of relapses in the previous two years or the presence of gadolinium-enhancing lesions.

## Conclusion

This study provides a realistic assessment of the challenges faced in developing prognostic models for MS patients and evaluating risk stratification [32]. Also, we observed that NNets yield better performance than classical regression models (simple logistics), indicating the usefulness of more advanced computational tools for handling complex datasets in multifactorial diseases. The variables selected by the model for inclusion exhibit good face validity and are at least partially independent. However, the current lack of a clear understanding of how much the short-term prognosis is informative about the long-term prognosis (decades later after disease onset) poses some limitations to our study. Nevertheless, the development of short-term disability classifiers can be of clinical value, such as helping in the prescription of DMD or improving clinical trial design and cohort recruitment [11,21,22,33]. Although significant challenges remain to be overcome before MS variables-based classifiers can be used in the clinical setting, these tools have the potential to improve patient care and can be customized for eventual clinical use [32].

## List of abbreviations used

MS: Multiple sclerosis; EDSS: Expanded disability status scale; MEP: Motor evoked potentials; CMCT: Central motor conduction time; SD: Standard deviation; A: Accuracy; S: Sensitivity; Sp: Specificity; PPV: Positive predictive value; NPV: Negative predictive value; ROC: Receiver operating curve; AUC: Area under the ROC curve; CIS: Clinically isolated syndrome; RRMS: Relapsing-remitting MS; SPMS: Secondary-progressive MS; MSFC: MS functional composite; MSSS: MS severity scale; TWT: Timed walked test; NHPT: Nine-hole peg test; PASAT: Paced auditory serial addition test; DMD: Disease modifying drugs; MRI: Magnetic resonance imaging; GM: Grey matter; WM: White matter; VBM: Voxel-based morphometry

## Competing interests

Authors have nothing to disclose. We certify that all our affiliations with or financial involvement, within the past 5 years and foreseeable future (e.g., employment, consultancies, honoraria, stock ownership or options, expert testimony, grants or patents received or pending, royalties), with any organization or entity with a financial interest in or financial conflict with the subject matter or materials discussed in the manuscript are completely disclosed.

## Authors' contributions

All authors read and approved the final manuscript. Author contribution: BB: study design, computational analysis, results analysis, manuscript preparation; LL: patient collection and analysis, data analysis, manuscript review; DGM: patients recruitment, test analysis, statistical analysis; JS: study design, MRI analysis, statistical analysis; JG: computational analysis; JA, OS, MB and UDC: MEP studies; GC: results analysis and discussion; PV: study design, patient recruitment, results analysis, manuscript writing.

## Pre-publication history

The pre-publication history for this paper can be accessed here:

http://www.biomedcentral.com/1471-2377/11/67/prepub

## Supplementary Material

Additional file 1 **additional methods and results**. it contains an extended description of methods, including description of the cohort, MEP, and development of computational classifiers. In addition, it also includes additional results, such as detailed MEP findings and attribute selection results of the computational classifiers. Click here for file
