# Supplementary material for: Computational classifiers for predicting the short-term course of Multiple sclerosis
Source: BMC Neurol. 2011 Jun 7;11:67. doi: 10.1186/1471-2377-11-67 (PMC3118106; doi:10.1186/1471-2377-11-67)
Supplement: Additional file 1 — additional methods and results. it contains an extended description of methods, including description of the cohort, MEP, and development of computational classifiers. In addition, it also includes additional results, such as detailed MEP findings and attribute selection results of the computational classifiers. [file 1471-2377-11-67-S1.DOC]

**Supplementary Methods**

***1. Clinical information of the test cohort***

We were interested in testing the associations in a cohort of MS patients in the early to intermediate phase of their disease, with no bias to select patients with clinical involvement of the motor pathways, higher disease activity or MRI lesion burden. The MS patients displayed mild to moderate disability and medium disease duration, as set out in the inclusion criteria. The cohort was composed of 16 clinically isolated syndromes (CIS), 26 patients with relapsing-remitting MS (RRMS), 3 with secondary-progressive MS (SPMS), 4 with primary-progressive MS (PPMS), and 2 with progressive-relapsing MS (PRMS). At the time of inclusion, 21 patients were receiving interferon beta and 2, chemotherapy (Mitoxantrone in one patient and Azathioprine in the other).

Of the original 51 patients, three were lost at the final assessment (month 24) but they were followed-up over a 21 month period. During the 2-year follow-up, the relapse rate was 1.31 (SD = 1.5) and accordingly, 21 patients did not suffer relapses (relapse-free patients), 10 had one relapse, 9 had 2 relapses, and 11 had at least 3 relapses. During the clinical follow-up 14 patients started new immunomodulatory therapy. In addition, 10 CIS patients converted to RRMS because they suffered a second relapse and 5 RRMS patients converted to SPMS. The change in EDSS during the 2 year period was 0.43 (SD 0.82; range 0-3.5). Disability progression was confirmed in 15 patients at 6 months and the final distribution of the disease subtypes at the end of the study was: 6 RRMS, 2 SPMS, 3 PPMS, 1 PRMS and 3 CIS).

# *2. Motor Evoked Potentials*

Using a circular magnetic coil (outer diameter, 13 cm), MEPs from the four limbs to the Transcranial Magnetic Stimulation of the motor cortex were recorded with a Medelec Synergy system (Viasys Healthcare, Oxford, UK) as electromyographic responses (EMG) through the adductor digiti minimi (ADM) and flexor hallucis brevis (FHB). The coil was positioned over the vertex to record the upper extremities and 4 cm anterior to the vertex to record from the lower extremities. MEPs were first obtained during muscle relaxation in order to obtain cortical motor threshold and resting MEPs, and then during slight voluntary tonic contraction of the target muscles (facilitation). We measured the following MEP parameters: motor threshold, area, amplitude, latency, central motor conduction time (CMTC), silent period (SP) with and without facilitation. MEP amplitudes and latencies were considered abnormal if they differed in > 2.5 SD from the normative database at our centre and the amplitude was also regarded as abnormal if there was a side-to-side difference of > 50%. CMCT is a useful measure to study central motor pathways, and it was calculated with the F-wave formula by determining the difference between the central latency and peripheral conduction time ^31^. The duration of the cortical SP induced by stimuli delivered at different intensities was measured as the difference between the TMS stimulus and the return of voluntary activity. For SP analysis, the measurements from each group of patients were compared with those from the control group. Evoked potential abnormalities were quantified for each limb according to a scale modified from Leocani et al. ^24^ (0 = normal, 1 = increased latency, 2 = increased latency plus decreased amplitude, 3 = absence of MEP response). The MEP score involving the 4 limbs was established within the range from 0 to 12. To analyze the effect of asymmetric disability, a Z score was created for each limb using the MEP CMCT (Z = (CMCT – mean)/SD). The worst Z score of the 4 limbs was selected and compared with the disability and disease subtype.

***3. Computational Classifiers***

The deterministic classifiers evaluated were 1) the naïve Bayes rule; 2) Simple logistic; and 3) Random decision-tree meta-classifier Bagging. The naïve-Bayes rule is a popular Bayesian method since it is a very fast and quite simple probabilistic approach that can achieve excellent results, outperforming more sophisticated classifiers for many datasets ^40^. Because attributes are treated as if they were completely independent, the addition of redundant ones skews the learning process. This can be ameliorated by using a subset of carefully selected attributes. Simple logistic is an up-to-date representative algorithm of logistic regression models that uses boosting to calculate the regressions (voting for classification or averaging for numeric predictions). The decision tree J48 classifier is an implementation of the C4.5 decision tree learner ^41^. C4.5 is based on the “divide and conquer” approach to the classification problem. The decision tree is a simple structure where non-terminal nodes represent tests of one or more attributes and terminal nodes reflect decision outcomes. In order to improve the performance of decision tree methods, we opted for the Bagging meta-classifier with a more powerful decision tree method, such as random forest. As a non-deterministic classifier we tested the neural network Multilayer Perceptron (MLP), a supervised feed-forward neural network that is trained with the backpropagation algorithm without making any assumption regarding the underlying probability density function.

***4. Attribute selection, patient distribution and validation procedure***

A more sensitive version of the Student’s t-test, known as a pair t-test, was used to determine whether the mean accuracy and mean kappa statistic across all datasets were significantly greater than the mean of another ^43^. Once obtained, each classifier was tested without further modification in the validation cohort in order to compare their yields in terms of diagnostic accuracy with the original test cohort.

As an input, the classifier received the following attributes: a) the clinical variables of MS subtype, sex, age, EDSS at study entry, motor function score of EDSS (MF), Multiple Sclerosis Functional Composite (MSFC), and motor scores of MSFC (TWT and NHPT); b) MRI variables: lesion volume in T1, gadolinium-enhancing lesion volume, grey matter and white matter volume; c) MEP variables including the CMCT (considering the worst performance of both sides for arms or legs), MEP score ^24^ (combined score from the 4 limbs), aggregated MEP score (MEP score grouped into 3 equal-range intervals), worst Z score from the 4 limbs, and abnormal MEP (presence or absence of at least one abnormal MEP in each patient). Some of these MEP variables were selected because they correlated with disability ^24^ and thus, they might offer good results to predict disease activity in our study. Six cases with no MEP potentials were assigned the same value as the case with the worst latency. As the primary end-point (output or dependent attribute), we considered the EDSS at the end of the study since this is the most common end-point in clinical trials assessing the efficacy of disease modifying drugs in MS. The EDSS was categorized in three intervals: 0-2.0; 2.5-4.5; > 5.0. Secondary outcomes were the absolute change in EDSS, disability progression (yes or no), and the occurrence of relapses by the end of the study (relapse-free status).
